# Supplementary material for: Are carbon emissions trading and green financial instruments synergistic? -Comprehensive quantitative research based on content analysis
Source: PLoS One. 2024 Mar 7;19(3):e0298601. doi: 10.1371/journal.pone.0298601 (PMC10919601; doi:10.1371/journal.pone.0298601)
Supplement: S1 Data — (ZIP) [file pone.0298601.s001.zip › Data/地级市Z_F工作报告.zip!/┴╔─■/│»╤⌠╩╨/└┤╫╘╣1⁄2╓┌║┼í╛┬φ┐╦╩2╛▌═°í┐_2005.docx]

各位代表：

我代表市人民政府向大会报告工作，请予审议，并请各位政协委员和其他列席人员提出意见。

一、2004年工作回顾

过去的一年，我们在省委、省政府和市委的正确领导下，认真贯彻党的十六大及十六届三中、四中全会精神，团结和带领全市各族人民，真抓实干，奋力拼搏，圆满地完成了市八届人大一次会议确定的2004年各项目标和任务，实现了振兴老工业基地的良好开局。

——国民经济持续快速健康发展，主要经济指标提前一年完成“十五”计划。预计实现生产总值152.8亿元，同比增长17.1%，按可比口径超出“十五”计划12.8亿元；其中工业增加值52.6亿元，超出“十五”计划16.6亿元。地方财政一般预算收入完成7.89亿元，增长37.1%，按可比口径超出“十五”计划5100万元。全社会固定资产投资完成64.6亿元，增长33.4%，四年累计超出“十五”计划68亿元。社会消费品零售总额实现56亿元，增长17.4%，超出“十五”计划4亿元。

——结构调整成效显著，三次产业全面发展。集中力量推进农业产业结构调整，畜牧业、保护地、林果业增加值占第一产业增加值的比重已达61.8%。畜牧业发展迅速，猪、牛、羊、禽出栏量分别达到108.5万头、14.9万头、110.9万只和2777.4万只，同比增长39.7%、30%、42.6%和23.2%。粮食生产喜获丰收，总产量达14.6亿公斤，是“十五”以来收成最好的一年。保护地建设取得新进展，新建大棚4万栋，总量达到31万栋。完成植树造林合格面积71.3万亩。农业产业化经营实现较大突破，规模以上龙头企业已达107户，其中新增销售收入3000万元以上企业18户。工业经济增势强劲，预计规模以上工业产值和利税分别实现169.7亿元和22.2亿元，增长44.2%和65.5%。汽车及汽车零部件、建材及建机、绿色食品加工“三大基地”和冶金、化工、纺织“三大行业”建设取得成效，产值分别实现35亿元和72亿元，拉动全市工业产值增长7个和23个百分点。新增销售收入超千万元、五千万元、亿元和2亿元企业分别达到66户、8户、9户和4户；一批新建和技改项目相继竣工投产。建筑业实现增加值8.4亿元，增长24.3%。第三产业持续发展，城乡市场购销两旺，繁荣稳定。旅游资源的普查和规划编制工作顺利进行。金融和保险业运行平稳，城市信用社组建一级法人社获得国家批准，并正式挂牌运营。税收征管得到有效加强，国地税征收总额达到21.4亿元，增长32.3%。民营经济高速增长，营业收入、实交税金分别实现205亿元和7亿元，增长31.6%和50.5%。三次产业在全面增长的基础上十年来首次呈现“二三一”排序。

——招商引资和外贸出口实现突破，开放型经济发展速度进一步加快。预计完成外商实际直接投资2000万美元，同比增长25%；新批外商投资项目27个，外资企业总数达到106个。完成域外招商引资32亿元，比上年增长1倍，域外投资超千万元的项目达34个。出口企业群体不断扩大，出口创汇实现1.05亿美元，增长125.6%。市经济技术开发区呈现较好发展势头，实现域外招商引资到位资金3.2亿元，又有9个投资千万元以上重点项目入驻开发区。县属项目区也各有发展，共入驻工业项目31个。

——各项改革全面推进，体制机制创新收到明显成效。国企产权制度改革已完成省考核任务量的95%，改制后启动生产的企业重现生机。农村税费改革继续深化，经过争取我市农业税全部免缴，农民实际负担水平降至零。土地使用和管理制度改革逐步深入，矿业秩序整顿取得初步成效。事业单位改革推进迅速，市县两级263个中介类和经营类单位划出事业单位管理序列；撤并乡镇事业单位1066个，精简编制员额5096名，精简率分别达到52%和54%。市县两级政府机构改革任务基本完成，行政审批制度改革进一步深化，新取消审批项目392个；保留审批项目进厅率、即时办结率分别达到95.4%和60.2%；全面清理行政事业性收费，取消市级收费项目73个。

——基础设施建设不断加快，城乡面貌发生崭新变化。大凌河城区段综合整治一期工程全面竣工，为古城朝阳增加了一道靓丽风景；北塔广场建成并向市民开放，使城市建设水平有了新的提升；北大街棚户区大面积改造，极大地改善了老城区居民的居住条件；东大桥主体工程、胜利桥明沟覆盖、火车站候车室改造、人行大厦、档案馆大楼、市二院门诊楼和站前广场、朝重广场建设全面竣工，中心城市增添了新的亮点。此外，新建和改造城市道路57条（处）、排水管线7.8公里、广场绿地13处，新增城区大树1.25万株、绿地48.8公顷、环城绿化面积2万亩，“城中村”改造工程启动实施，垃圾无害化处理厂建成投入使用，中心城区空气质量又有新改善。县城及重点小城镇建设进一步加快，北票采煤沉陷区治理工程高标准规划建设，超额完成省政府下达的任务；各个县城的基础设施不断完善，中心城镇面貌发生新的变化，辐射功能进一步增强，有效地拉动了县域经济的发展。全市新铺黑色路面533公里，路基改造1228公里，120个村通上了油路。山区建设和农田基本建设扎实推进。交通运输、邮政、通信能力明显增强。

——人民生活水平显著提高，向群众承诺的实事得到全面落实。预计实现城镇居民人均可支配收入5683元、农民人均纯收入2415元，同比分别增长10.4%和31%；城乡居民储蓄余额达191.1亿元，增长13.3%。社会保障工作得到加强，养老、医疗和失业保险参保人数分别达到20.8万人、23.8万人和23.4万人，有14.3万名城镇困难群众得到最低生活保障，基本实现了应保尽保。积极开发就业岗位，全年安置就业和再就业5.08万人，城镇登记失业率控制在6.46%。全年完成劳务输出32万人次，实现经济收入13亿元。扶贫开发和扶贫帮困工作又有新进展，45万农户拥有稳定收入项目，395个村实现脱贫，900户贫困户实施移民搬迁，5.62万名困难群众得到临时救助，8.2万名贫困学生得到资助。市政府在八届人大一次会议上承诺的九件实事全部落实：一是企业最低工资标准由240元提高到350元；二是城镇居民最低生活保障标准普遍提高14元，人均每月实发73元，达到全省平均水平，其中4.72万名无劳动能力的低保对象提标幅度达20%以上；三是市本级退休人员已纳入基本医疗保险范围；四是多渠道筹措供暖基金，使2040户城市特困家庭温暖过冬；五是着力解决大龄人员就业问题，安置“4050”人员就业4515人；六是制定了失地人员参加养老保险实施办法；七是改造新建安居房30万平方米，3700多户居民喜迁新居；八是采取多种措施帮助农民工清偿拖欠工资690万元；九是在农村新建224处基本饮水井和防氟井，均已投入使用。此外，各级政府在确保公教人员工资按时足额发放的同时，还多方筹措资金，兑付了2001年度以前拖欠的公教人员标准工资1.48亿元，占陈欠总额的62%。

——社会事业蓬勃发展，精神文明和民主法制建设不断加强。科技事业快速推进，启动实施了振兴老工业基地科技行动计划，争取国家和省各类科技专项38项，签定各类合作合同82项；5户企业进入市高新技术园区；全市高新技术产值实现27.1亿元，增长29%。教育优先发展进一步落到实处，改造农村中小学危房9.22万平方米，开工兴建九年一贯制学校13所；城区小学大班额问题得到缓解；新增普通高中校舍面积9.4万平方米，普通高中扩招比例达22.1%；职业教育规模逐步扩大，实际招生比上年增加21%。文化事业再结硕果，话剧《凌河影人》获中国文华大奖和第七届中国艺术金奖，“凌河之夏”等各种群众文化活动丰富多彩，文化市场通过依法治理得到进一步净化。体育基础设施逐步完善，市体育场、全民健身广场建成投入使用；成功举办了第六届全运会，全民健身活动日益普及，竞技水平稳步提升。医疗卫生事业健康发展，疾病防控有效加强，药品市场管理进一步规范。人口和计划生育工作不断加强，各项控制目标全部完成省下达计划。民族地区经济文化实现新发展，民族政策得到较好落实。民兵预备役、人防等工作取得新成绩，人防指挥中心竣工落成。围绕打造“信心朝阳”、“学赤峰、促振兴”等主题，开展各类精神文明创建活动，全民思想道德素质进一步提高，发展信心明显增强。军民共建活动广泛深入，军民情谊更加深厚。市政府自觉接受人大及其常委会的依法监督和政协的民主监督，认真听取各民主党派和工商联的意见和建议，进一步加强与工会、共青团、妇联等群众团体的联系。办理人大代表建议56件，政协提案144件，办复率达100%，满意率和基本满意率达97%以上。强化市民投诉办理，建立政府与企业家联系的绿色通道，设立行政效能监察举报专线，实施行政过错责任追究，从严查处违法违纪案件，软环境建设取得初步成果。扩大政务公开领域，严格行政执法监督，坚持对重大决策和事关公众利益的重大事项、重点项目实行专家论证、广泛听证，多方征求意见，政府民主法制建设得到加强。巩固和发展基层民主，圆满完成了第八届村民委员会换届选举工作。强化治安防控体系建设，严惩各类刑事和经济犯罪，着力打造“平安朝阳”。高度重视群众来信来访，认真解决群众合理诉求，及时处理各种矛盾和纠纷，保持了社会的基本稳定。

各位代表！过去的一年，我们在发展中遇到的困难比预料的多，取得的成效比预期的好。这是全省上下关心朝阳、支持朝阳，省委、省政府和市委正确决策和坚强领导的结果，是全市各级人民代表大会及其常委会和人民政协监督支持的结果，是全市各级干部和各族人民团结奋斗、开拓进取的结果。在此，我代表市人民政府，向奋斗在各条战线的广大工人、农民、知识分子、各级干部，向全社会的劳动者和建设者，向对政府工作给予充分理解、热情支持的人大代表、政协委员、各民主党派、工商联、人民团体和各界人士，向关心、支持朝阳发展的中省直单位、人民解放军和武警驻朝官兵，致以崇高的敬意和衷心的感谢！

成绩为乘势而上奠定了坚实基础，也坚定了全市上下加快发展的决心和信心。但我们也清醒地认识到，过去一年经济的快速增长带有很大的不稳定性和不平衡性，经济社会发展中还存在许多深层次矛盾。一是经济结构性矛盾仍十分突出，经济增长方式没有实现根本转变，三次产业的“二三一”排序尚处于不稳定状态，骨干产业、骨干企业和骨干基地发育不快，第三产业发展十分缓慢。二是体制机制创新任务仍非常艰巨，国企改制任务尚未彻底完成；事业单位人事分配制度改革刚刚启动，尚待深化；民营企业“两权分离”和投资主体多元化及建立现代企业制度远未破题；其他方面改革与预期也存在较大差距。三是固定资产投资水平与加快朝阳发展、振兴老工业基地的需求不相适应，金融机构投放贷款能力有限，民间资金转化为民营资本的途径不多，具有牵动力的大项目少的局面没有明显改变。四是城乡群众收入仍处于较低水平，农村贫困群体仍然较大，城镇低保实际保障水平不高，就业和再就业工作面临较大压力。五是城镇化水平低，规划和管理水平不高，基础设施建设滞后，集聚产业和吸纳人才的功能较差。六是社会事业发展投入不足，人才总量不足和结构不合理问题特别突出。七是随着各项改革的推进和利益格局的调整，社会不稳定因素增加。各级政府承受着推进发展、深化改革、确保稳定的巨大压力，经受着执政能力的严峻考验。然而在压力和考验面前，政府机关的一些部门和单位一心一意谋发展、围绕中心抓落实的自觉意识不强，工作效率和服务水平不高；一些干部机遇意识、责任意识和危机意识不强，思想不够解放，工作作风飘浮，知识和能力不适应、不符合履行岗位职责的要求；有的部门和行业消极腐败现象和不正之风尚未得到有效遏制，违法行政、行政不作为以及伤害管理服务相对人合法权益的现象屡有发生；软环境治理任务特别繁重；政府职能转换还不到位，等等。新的一年里，我们要高度重视上述问题，采取有力措施认真加以解决。

二、2005年主要工作任务

各位代表，2005年是全面推进老工业基地振兴的关键一年。做好今年的工作，对于全面完成“十五”计划目标，为“十一五”经济社会的更快发展奠定坚实基础，意义十分重大。

总体要求是：高举邓小平理论和“三个代表”重要思想伟大旗帜，深入贯彻党的十六大和十六届三中、四中全会以及省委九届八次全会、市委七届八次全会精神，以科学发展观为统领，以推进“一主三化”为主攻方向，以结构调整为主线，以改革开放为动力，以项目建设为支撑，以壮大企业和搞好农业产业化经营为主要抓手，推动城乡经济又快又好地发展，着力构建和谐社会，促进社会全面进步，加快朝阳老工业基地调整改造进程。

主要预期目标是：生产总值增长15.5%，地方财政一般预算收入增长15.6%，全社会固定资产投资增长30%，城镇登记失业率控制在6%以内，劳务输出28万人次，域外招商引资45亿元，城镇居民人均可支配收入和农民人均纯收入分别达到6250元和2660元，各项社会事业与经济建设协调发展、同步提高。实际工作中，我们将向更高目标努力。

为了确保实现上述目标，我们要全力做好以下六个方面重点工作：

（一）以结构调整为主线，以项目建设为支撑，促进国民经济快速、健康、协调发展

全力以赴抓好项目建设。坚持不惜血本引进项目，不遗余力开发项目，穷尽招法建设项目。进一步发挥政府、企业等多个积极性，层层落实项目工作责任制，全年确保实施投资超千万元的重点项目200个以上，新增重点前期储备项目300个以上。凌钢新建200万吨钢和燕山湖发电项目争取年内获得核准并开工建设。京四高速公路朝阳段全面开工建设，朝赤高速公路朝阳段控制性工程启动实施；充分发挥高速公路交通优势，规划建设各具特色的经济开发带。大凌河城区段二期改造、东大桥、北大桥、农村公路网1000公里路基建设、“阎水北调”供水工程等一批基础设施项目确保完成计划目标。浪马新增20万套子午胎、朝纺新增4万锭和1000万件针织内衣、九道岭75万吨原煤、电源公司18万台一体化电源、建平陶瓷工业园16条生产线以及飞马铸造、天亿制药等项目确保年底前竣工投产；朝柴10万台柴油机、思益汽车零部件和组装车、凌钢保国铁矿扩建以及兰凌、东鑫、四合当、群峰4个日产5000吨水泥项目年内争取实现突破。千方百计搞好项目筛选、论证，加大储备、包装力度，特别要发挥各个县（市）区的资源、产业等优势，争取更多的项目挤进老工业基地调整改造和省“软贷款”项目盘子。

积极推进工业结构调整与产业升级。围绕“三大基地、三大行业”建设，以接长产业链条、提高产品附加值和产业关联度为主攻方向，加快调整工业结构，保持工业经济快速增长势头，确保工业增加值增长20%以上。用好用足各项扶持政策，加快冶金、机械、化工、纺织、医药、建材等重点行业的技术改造。抓好骨干企业梯队培育规划特别是“十企一园”发展规划的落实，围绕骨干企业和优势产品搞好协作配套，积极帮助企业做大做强，力争新增销售收入超5000万元企业11户、超亿元企业5户。市县两级政府要搞好产业规划，强化调控手段，引导各类资本更多地投向低污染、低能耗、高技术含量、高附加值的项目，支持开发建设具有较强产业牵动力、市场竞争力的大项目，发展循环经济、配套经济和扩大就业的产业，坚决制止低水平重复建设。

进一步加快农业结构调整步伐。围绕绿色食品加工基地建设，朝着生产工厂化、加工规模化以及形成最终产品的方向，加快农业产业化推进步伐，重点新上一批农副产品深加工项目，培育一批主营产品优势明显、成长潜力较大、带动农民增收能力较强的龙头企业。强力推进畜牧业发展，采取综合扶持措施，引导发展畜牧业大场大户，全面实施畜牧小区产能翻番，扩大农户庭院养殖，加快良种扩繁步伐，增加粮饲兼用玉米种植面积，提高畜产品商品量和附加值，畜牧业产值占农业总产值的比重力争达到50%左右。继续营造保护地生产的规模、质量和市场优势，突出搞好新品种引进，全年新建大棚4万栋。加快推进生态朝阳建设，巩固发展封山禁牧成果，大力发展林下经济，完成人工造林和封山育林75万亩、农村能源四位一体生态农业模式示范户1万户。着力搞好无公害、绿色、有机农产品的标准化生产，加快认证速度，提高绿色食品产出率和加工率。规划建设好农产品批发市场，培育发展农民合作经济组织，提高社会化服务水平。坚持不懈地搞好山区建设和农田基本建设，不断提高农业机械化水平，加大农业减灾防灾设施投入力度，提高农业抗灾能力和综合生产能力，完成水土保持综合治理面积80万亩。加快高效农业配水工程建设，有计划地搞好河道治理，加强城乡的水资源管理。

加快发展现代服务业。积极推进金融业改革和发展，支持各金融单位灵活运用各种手段，为地方经济发展服务。加大城市信用社和农村信用社增资扩股力度，鼓励社会自然人、企业法人通过合法规范渠道入股，在组建城市信用社股份有限公司的基础上，进一步做大做强地方商业银行；切实搞好农村信用社改革，大力清收不良贷款，逐步实现向农村合作银行过渡。加快现代商业设施建设，大力发展代理、物流、中介、超市等新型业态，加快提档升级步伐；结合朝百大楼和工人文化宫改造，进一步完善新华广场商业区功能，增强辐射作用。积极发展覆盖广、多层次、便利化的社区服务业和会计、律师、咨询、保险等现代服务业。促进房地产市场持续健康发展。依托红山文化、三燕文化及古生物化石等优势旅游资源，加快旅游景点、旅游产品和旅游市场开发，力争使朝阳早日跨入全省重点旅游城市行列。强化市场监管，进一步整顿和规范市场经济秩序，严厉打击食品、药品、保健品、农业生产资料等领域的制假售假行为，切实保障消费者合法权益。

（二）进一步扩大开放，广泛开展全民创业，不断拓展经济发展空间

加大招商引资和出口创汇力度，努力构建全方位的对外开放格局。认真研究全球产业结构调整和国际资本流动规律，密切关注沿海经济发达地区产业技术升级特点和趋势，依托我市优势资源、现有企业基础，建立项目承接、引进和推进机制，通过项目的引进，提高我市经济的外向依存度。创新招商方式，强化招商手段，灵活开展以商招商、亲情招商、代理招商、网上招商和定向招商，积极拓展对外联系渠道，增强招商引资的针对性和实效性。把开放范围扩大到商业、文化、科技、教育、卫生、体育、旅游、城市基础设施等各个领域，在引进资金项目的同时，积极引进经营理念、优秀人才和先进技术，促进产业技术升级和管理创新。进一步完善市经济技术开发区和各县（市）区项目区管理体制，实现基础设施建设、形象塑造、招商引资、产业聚集、管理创新和经济总量新突破，市开发区招商引资和固定资产投入要均完成4亿元以上，实现经济总量增长40%以上。继续保持外贸出口的良好态势，在抓好出口大户和大宗产品出口的同时，积极引导各类有条件的企业开拓国际市场，培育新的出口增长点。强化实施“走出去”战略，搞好扶持和服务，推动有实力的企业参与国际经济技术合作，重点开拓境外、域外建筑市场。全年实际利用外资、出口创汇均增长25%以上。

积极开展全民创业，大力发展民营经济。朝阳加快发展的根本途径在于激发全民自我发展的动力。在全市范围广泛开展全民创业活动，动员和组织全市广大干部群众积极投身到全民创业中去，掀起全民竞相创业的热潮，大力培育各个领域、各个层次、各种类型的创业主体。把营造宽松的政策环境作为重中之重来抓，除国家明文禁止外，所有领域都对民间资本、民营企业和社会自然人开放，支持民营企业创办投资公司、担保机构，参与基础设施和公用事业建设以及投资教育、文化、卫生、科技、旅游等产业；进一步简化注册登记程序，降低准入门槛，鼓励各级机关干部离岗领办创办经济实体，允许事业单位科技人员从事第二职业进行创业；加快建立和完善面向中小企业和全民创业的贷款担保体系，建立健全民营企业信用评估制度和办法，认真解决好全民创业和民营企业用地难、融资难、担保难等问题。通过各种有效措施，让所有有志者无障碍地投身创业，让各种发展要素顺畅地在朝阳集聚，营造人人争做全民创业参与者、服务者、支持者和推动者的良好局面，形成尊重纳税人、争当纳税人的社会风尚，使全民创业成为新时代朝阳人的人文精神和内在追求，推动全市民营经济总量大幅度提升，确保民营经济营业收入、入库税金均增长30%以上。

（三）深化各项改革，创新体制机制，为经济加快发展提供动力支撑

大力推进企业改革。进一步完善各项配套政策，做好国企改制收尾和遗留问题处理工作，朝重、长征轮胎两户企业的改制要尽快取得突破，市直建筑、商业、医药及其他非工口企业改制工作年内确保全面完成。大力发展混合所有制经济，积极引导已改制企业和民营企业通过增资扩股、合资合作、资产重组等方式进行股份制改造，加快建立和完善现代企业制度，完善公司法人治理结构，年内力争有50%左右的民营企业实现投资主体多元化。充分发挥市场机制作用，全面放开城市供水、供气、供热、公共交通、污水处理、垃圾处理等行业市场。

积极推进农村改革。继续深化国土资源管理体制改革，落实最严格的耕地保护制度，切实保障农民利益；积极推进农村集体建设用地使用权依法流转，鼓励土地使用者以出让、租赁、作价入股等方式进行土地经营。巩固农村税费改革成果，做好配套改革的跟进和衔接工作，逐步化解乡村债务，及时研究解决改革后出现的新矛盾、新问题，确保农民负担不反弹，确保基层干部工资及时足额发放，确保基层政权有效运转。加快推进农村小型水利设施、农林场产权制度改革和粮食流通体制改革。

继续推进财税改革。在做好市本级编制部门综合预算的同时，全面深化县（市）区的预算编制制度改革。进一步扩大国库集中支付范围，规范政府采购和非税收入收支两条线制度。搞好财力资源整合，不断完善分税制财政管理体制，进一步调动县乡政府发展经济、培植财源的积极性。加强税收征管和税式支出管理，积极做好增值税转型试点工作，认真落实各项扶持就业、促进老工业基地振兴的税收政策措施。

全面推进事业单位改革。乡镇事业单位改革在完成人员定岗的基础上，做好人员分流工作；市县两级事转企单位在年内完成产权制度改革任务；行政管理类和部分社会公益类事业单位进一步加快进行职能整合，搞好用人制度、分配制度改革。大力发展民办教育，加快推进卫生、文化、体育等事业单位产权制度改革步伐，努力形成多元投资、公平竞争、规范有序的社会事业发展格局。

（四）进一步提高城镇化水平，扶持和壮大县域经济，实现城乡统筹协调发展

加快发展中心城市。做大中心城市，对加快城镇化步伐、推动区域经济发展，具有巨大的辐射带动作用。今年，中心城市要以凤凰组团基础设施建设为重点，规划拓展东部发展空间；以加速开发区和冶金工业园区建设为重点，向西北、西南方向推进；以朝阳县县城搬迁为重点，使城市南缘向柳城方向扩展，积极构建50平方公里、50万人口的城市框架。加快旧城区、城中村改造和北大街工程建设步伐，加大破损路、断头路、泥土路改造力度，启动建设市古生物化石地质公园、污水处理厂、客运中心、珠江路立交桥和凤凰山综合整治工程，全面完成凌凤大街改造，提升七道泉子和西大营子两个出口的整体形象。以集约用地、依法用地为目标，进一步规范城市土地出让工作，增加土地收益。强化城市管理，建立和完善城市管理长效机制，加大综合执法力度，着力解决破坏绿地树木、占路经营、烟尘污染、噪声扰民、私搭乱建等问题，实现建管水平的同步提升。

大力推进县城和重点小城镇建设。加快县城和重点小城镇建设步伐，是提高城镇化水平的重要内容和重要支撑。坚持高起点规划，科学布局，增加投入，拓展县城和重点小城镇发展空间；深化户籍制度、就业制度和社会保障制度，积极探索“以土地换社保”等有效途径，尽快消除阻碍城镇化发展的体制性障碍；进一步完善城镇功能，全力搞好北票采煤沉陷区治理、朝阳县县城基础设施一期工程、喀左县双桥大街改造工程、建平县新水源建设、凌源市集中供热三期工程等项目；加快县城和重点小城镇主要街路、出入口、城乡结合部的净化和绿化，增强吸纳农村人口、带动经济发展的能力，推进人口向城镇集中、企业向园区集中、土地向“庄园”集中。

激发县域经济活力。坚持从各地实际出发，以“一主三化”为主攻方向，发挥各自优势，发展市场需求的特色产业，确保县域经济主要指标增幅高于全市平均水平。完善政策措施，实施简政放权，在招商、立项、审批等方面赋予县级政府更大的发展自主权和决策权。市政府对各县实行共享税种增量全部返还的扶持政策，并且从增量财力中安排专项资金，用于鼓励支持县域经济发展。扶持优势产业，创新考评办法，实行分类指导，切实把“叫真、较劲、叫板”落到实处，努力在全市形成县域经济发展的良性激励机制。积极支持凌源市搞好全省县域经济发展试点工作。

实现人口、资源、环境的协调发展。认真落实计划生育工作责任制，进一步稳定低生育水平，开展出生性别比失调的专项治理，严格执行人口失控责任追究，从严查处超计划生育和瞒漏报行为。人口出生率控制在10‰以内，计划生育率稳定在98%以上。强力开展矿业秩序综合治理整顿，花大力气解决乱采滥挖、污染环境、浪费资源等突出问题，最大限度地延长产业链条，促进矿产资源深度开发和综合永续利用，引导企业把积累用于提高技术水平、增强发展后劲上。进一步保护和开发利用好古生物化石资源。加强环境保护与污染治理，不断改善环境质量，确保市区空气二级指标天数达到80%以上。不断提高安全经济供电水平，加快推进铁路、交通、邮电、通信等事业发展。

（五）坚持以民为本，全力提高人民群众生活水平

集中力量打好城乡扶贫攻坚战。坚持开发式扶贫为主、开发式扶贫与救助式扶贫并举的方针，以贫困村为主战场，以落实户有稳定收入项目和加快农村劳动力转移为主要途径，按照“分解到年、整村推进、落实到户、因类治贫”的原则，进一步完善扶贫规划，加大社会扶贫工作力度。重点抓好项目入户工作，确保新增拥有稳定收入项目的农户达5万户以上；根据贫困成因，分别采取救灾、搬迁、治愚、医病、助学、帮残等多种措施，实行有针对性的帮扶，切实帮助农村贫困人口提高收入水平，实现温饱。以棚户区改造为重点，打好城市扶贫攻坚战，力争两年内消灭中心城市和县城所在地的棚户区。建立健全城乡困难群众社会救助体系，解决好城乡人民群众出行难、安居难、看病难、上学难等实际问题。继续做好防氟改水工作，搞好城市供暖，提高物业管理的质量和水平。

继续抓好就业和社保工作。把增加就业、促进再就业作为政府工作的优先目标，放在更突出位置。拓宽就业渠道，在统筹发展、结构调整中实现就业，在政策扶持、发展经济中促进就业，在转变观念、全民创业中增加就业，全年实现就业和再就业4.5万人以上。要把劳务输出作为一项战略任务和重要产业，进一步加快劳务派遣组织和中介机构建设，搞好外出务工人员的组织、指导、培训、维权等全程服务，推动劳务输出工作实现新突破。依法规范清理劳动关系，进一步扩大劳动合同制度覆盖面。认真开展就业和社会保障状况普查检查，以吸纳混合所有制、非公有制从业人员和灵活就业人员参保为重点，不断扩大社会保险覆盖面。进一步完善社保体系和社保网络建设，加大各类保险费的征缴和筹集力度，严格支出管理，确保各项社会保险费按时足额发放。健全城镇居民最低生活保障制度，加快完善低保对象动态管理办法，将符合条件的城镇居民全部纳入低保范围。全面实行住房公积金制度，切实保障职工的合法权益。

（六）全面推进各项社会事业发展，加强精神文明和民主法制建设，致力营造改革和发展的良好环境 

推进科教兴市和人才强市战略。深入实施振兴老工业基地科技行动计划，加快市高新技术园区建设，提升创新能力、孵化能力和辐射能力；加强与大专院校、科研院所、海外学子的合作，积极引进、推广国内外高新技术和先进适用技术。继续完善以县为主的农村义务教育管理体制，突出抓好农村中小学校布局调整和控辍保学，基本完成危房改造任务，巩固提高“普九”成果；整合大中专院校教育资源，高标准搞好7所普通高中建设，确保完成扩招任务；大力强化职业技术教育，特别是着眼于为劳务输出服务，积极开展订单培训；引入竞争机制，规范中小学教学人员招聘录用工作。紧紧抓住培养、吸引、留住和使用四个环节，进一步创新和完善人才管理体制和机制，努力为朝阳老工业基地振兴提供人才支撑。

促进卫生、文化、体育等各项社会事业发展。加强公共卫生服务体系建设，提升医疗服务质量和突发性公共卫生事件应急能力；加快农村卫生布局调整和资源整合，建立农村医疗救助制度，提高农民医疗保健水平。繁荣文艺创作，壮大文化事业，发展文化产业；加强文物保护和开发利用，规范和净化文化市场。大力普及全民健身活动，不断提高竞技体育水平，举办好首届全民体育健身节。坚持正确的舆论导向，扩大广播、电视节目覆盖，加快新闻出版等事业发展。继续大力支持民族地区经济和社会事业发展，依法管理宗教事务。广泛开展全民国防教育，进一步抓好双拥共建、优抚和人防工作。做好妇女、儿童、老龄和关心下一代工作，积极发展残疾人事业，启动第二轮地方志编撰工作。做好国民经济和社会发展第十一个五年计划纲要编制工作。

切实加强精神文明和民主法制建设。全面贯彻落实《公民道德建设实施纲要》，围绕打造“信心朝阳”，在继续把“学赤峰、促振兴”活动引向深入的同时，广泛开展“产业文化进家庭”、“行业形象万人评”、“图强致富文明带”、“联手合力育新风”以及“文明城市七整治”等群众性精神文明创建活动，努力形成发展、创新、团结、向上的社会氛围。更加自觉接受人大及其常委会的法律监督、工作监督，主动报告各项工作，认真落实执行人大及其常委会的决议决定，高度负责地办理好人大代表议案和建议，积极配合人大常委会及人大代表的“三查（察）”活动。通过各种形式和途径接受政协的民主监督，进一步实现接受监督制度化、民主协商经常化。主动听取各民主党派、工商联的意见和建议，发挥工会、共青团、妇联、科协等人民团体的作用。加强社区民主建设，健全村民自治，认真抓好乡镇政务公开和村务公开。深入开展法制教育，提高全民法制观念和法律素质，带动全社会形成学法、知法、守法、用法的良好风气。

做好安全生产和社会稳定工作。进一步落实安全生产责任制，加强对重点行业、重点领域和重点企业的日常监管，加大投入力度，及时排除隐患，坚决遏制重特大安全事故的发生。深入开展“矛盾纠纷大排查、大调处”活动，高度重视群众来信来访，及时解决群众的合理诉求，最大限度地把各种社会矛盾纠纷解决在初始阶段、化解在基层。加强社会治安综合治理，构建全方位、多层次的社会治安防控体系，保持打击各种刑事犯罪和经济犯罪的高压态势，铲除“黄赌毒”等丑恶现象。深入开展城乡交通秩序大整顿活动，为人民群众的生产生活创造安全稳定的社会环境。

加强软环境建设。深化行政审批制度改革，继续削减审批事项，编制和实施好审批事项办理流程，全面实行行政许可事项办理“提速工程”，真正实现快捷、高效、便民。深入开展“选优评差”活动，切实加大涉软案件查处力度，确保涉软案件查处工作在年内取得明显成效。加强行政效能监察，切实提高行政效率和服务水平，努力让投资者和创业者满意、人民群众顺心。大力提高全社会的环境意识，在营造亲商安商的人文环境上实现突破，努力形成“人人关注软环境，人人建设软环境”的浓厚氛围。

三、加强政府自身建设

面对新形势、新任务，我们既要坚定信心，乘势而上；又要居安思危，增强忧患意识，自觉地加强执政能力建设，实现好、维护好全市人民的根本利益。要把工作的着眼点聚焦到加快发展上，把工作的着力点集中到真抓实干、狠抓落实上，努力把各级政府建设成为适应振兴老工业基地要求的、对人民高度负责的务实、高效、廉洁、法制政府。

对人民高度负责，要求我们必须加强学习、提高素质。各级公务员要加强新知识、新理论和法律法规的学习，不断提高思想政治素质、业务素质和驾驭市场经济的能力，努力造就一支政治坚定、业务精湛、作风正派的高素质公务员队伍。要按照市委的统一部署，在政府机关深入开展保持共产党员先进性教育活动，教育引导各级公务员树立正确的理想信念、高尚的道德情操和与时俱进的思想观念，坚持执政为民、用权为公，不断提高履行职责的能力。

对人民高度负责，要求我们必须提高科学、民主、依法行政的能力。要建立政务信息公开、重大决策社会公示、听证和专家论证制度，推行政府新闻发布制度，深入实施政府与群众“连心桥”工程，建立和完善集体决策机制。深入学习和贯彻《全面推进依法行政实施纲要》，提高全体公务员的法制意识、法律素质和依法行政能力。狠抓行政执法队伍建设，采取综合措施提高执法服务水平；严格执法监督，将各项法律法规严肃公正地执行到位。

对人民高度负责，要求我们必须提高抓落实的效率和本领。要坚持“发展是第一要务、落实是第一基本功”的理念，做出的决策，定一项是一项，项项算数；承诺的事情，干一件成一件，件件落实。大幅度精简会议、文件，严格控制不必要的检查、评比、达标等活动，继续推行“无会月”制度，使各级干部有更多的时间深入基层、解决实际问题。进一步强化督促检查，完善约束激励机制，把各项工作要求落到实处。

对人民高度负责，要求我们必须提高清廉治政的自觉性。要坚持标本兼治、综合治理、惩防并举、注重预防的方针，建立健全与社会主义市场经济体制相适应的教育、制度、监督并重的惩治和预防腐败体系。不断增强廉洁从政的意识，严格遵守国家公务员条例，认真执行党风廉政建设责任制及廉洁自律各项规定。采取更为严厉的措施，加大对违法违纪案件的查处力度，惩治以权谋私、权力寻租行为，纠正部门和行业不正之风，狠刹奢侈浪费歪风，维护公平、公正的行政环境，以反腐、倡廉、治贪的实际成果取信于民。

对人民高度负责，要求我们必须以民为本，全心全意为民谋利。为人民掌好权，靠人民行好政。时刻牢记为民宗旨，把群众需要作为第一信号，把为民造福作为第一追求，真心诚意地为群众解难题、排忧愁。根据广大群众的要求，立足于现实条件和能力，市政府确定在新的一年里，办好9件与人民群众息息相关的实事：①建立和实施农村最低生活保障制度；②启动北票市农村新型合作医疗试点；③采取综合措施，扶持现有20.6万名处于国家标准以下的贫困人口基本解决温饱问题；④改造城市棚户区30万平方米；⑤多渠道筹措资金1亿元，兑付拖欠的公教人员标准工资；⑥新安置“4050”人员就业2000人以上；⑦启动建设朝阳大学；⑧完成市疾控中心搬迁改造和传染病院异地新建；⑨建设市图书馆、体育馆、第七中学。

各位代表！朝阳是一个充满生机和活力的地方，是一个能干事、能干大事、能干成大事的地方。勤劳智慧的朝阳人民创造了昨日的辉煌，也一定能够赢得更加美好的明天。我们既要有忧患意识、危机意识，更要有自信心，有一种蔑视困难、战胜困难的勇气。让我们在省委、省政府和市委的坚强领导下，以只争朝夕、奋发有为的精神状态，脚踏实地、求真务实的工作作风，把人民的期盼变成前进的动力，把历史的使命化为实干的行动，与时俱进，开拓进取，励精图治，团结奋斗，在振兴朝阳老工业基地、全面建设小康社会的伟大征程上，迈出新步伐，再跨新台阶！
